# Synchrotron Radiation–Excited X-Ray Fluorescence (SR-XRF) Imaging for Human Hepatocellular Carcinoma Specimens

**DOI:** 10.3390/cancers18020311

**Published:** 2026-01-20

**Authors:** Masakatsu Tsurusaki, Keitaro Sofue, Kazuhiro Kitajima, Takamichi Murakami, Noboru Tanigawa

**Affiliations:** 1 Department of Radiology, Kansai Medical University Medical Center, Osaka 570-8507, Japan; 2 Department of Radiology, Kobe University Graduate School of Medicine, Kobe 650-0017, Japan; ksofue@med.kobe-u.ac.jp (K.S.); murataka@med.kobe-u.ac.jp (T.M.); 3 Department of Radiology, Hyogo Medical University Faculty of Medicine, Nishinomiya 663-8501, Japan; kazu10041976@yahoo.co.jp; 4 Department of Radiology, Kansai Medical University, Osaka 573-1191, Japan; tanigano@hirakata.kmu.ac.jp

**Keywords:** copper, trace elements, liver neoplasms, electrons, zinc, hepatocytes, tumor microenvironment, magnetic resonance imaging, electron probe microanalysis, spectroscopy, electron energy loss

## Abstract

Background/Objectives: Trace metals, including copper (Cu) and zinc, are associated with the development and prognosis of hepatocellular carcinoma (HCC). However, their interference with magnetic resonance imaging (MRI) limits their use as potential biomarkers. This study investigated the usefulness of Synchrotron Radiation–excited X-ray Fluorescence (SR-XRF) imaging in studying the distribution of trace metals in HCC. Methods: This case–control study analyzed 33 specimens from 32 patients with HCC who underwent surgical resection (*n* = 29) or biopsy (*n* = 3) at Kobe University Hospital between December 1999 and November 2002. The findings of SR-XRF were compared with those of MRI and histopathology. Results: SR-XRF provided two-dimensional mapping of trace metal distribution with high spatial resolution (1.0 µm). The mean tumor-to-liver ratio (TLR) of Cu content was significantly higher in well-differentiated HCCs than in moderately and poorly differentiated HCCs (*p* < 0.05). Moreover, the mean TLRs of Cu content were significantly higher in high-intensity lesions than in iso- or low-intensity lesions on T1-weighted imaging (*p* < 0.05). Conclusions: This study supports previous evidence of the involvement of Cu in HCC development, suggesting its potential as a clinical biomarker for diagnosis and disease progression. Additionally, the results demonstrate that SR-XRF has potential for clinical application due to its ability to map trace metal distribution at high resolution. These findings suggest, rather than demonstrate, the association among Cu accumulation, tumor differentiation, and MRI signal characteristics.

## Simple Summary

This study investigated the usefulness of SR-XRF to examine trace metal distribution in hepatocellular carcinoma by comparing the distribution of copper (Cu) and zinc in SR-XRF with histopathology and magnetic resonance imaging. SR-XRF provides important insights into the underlying pathophysiological processes of tumor formation and progression. The findings demonstrate a relationship between Cu accumulation and tumor differentiation and T1WI high signal intensity, which underscores the potential for the Cu tumor-to-liver ratio to serve as a marker of differentiation, contributing to diagnosis, prognosis estimation, and interpretation of stepwise tumor progression, ultimately bridging imaging, pathology, and elemental omics. This method integrates the requirements of “distribution” and “quantification” by allowing a non-destructive, multi-element, high-sensitivity mapping, which is difficult to achieve with conventional methods, thereby enabling evaluation of the boundary between the tumor and the surrounding liver, as well as tumor heterogeneity.

## 1. Introduction

Liver cancer is the sixth most common cancer and the third leading cause of cancer-related deaths worldwide, with men having a higher risk than women [1]. Hepatocellular carcinoma (HCC), the primary cancer of hepatocytes, accounts for more than 80% of primary liver cancer cases globally [2].

The incidence and mortality of HCC have been increasing in North America and several European regions, and its main risk factors include chronic hepatitis C virus (HCV) or hepatitis B virus infection, heavy alcohol consumption, diabetes, and possibly non-alcoholic fatty liver disease [3].

HCC is highly heterogeneous and has a poor prognosis, with a 5-year survival rate of <20%. Currently identified prognostic and diagnostic biomarkers include alpha-fetoprotein (AFP), AFP-L3, glypican-3, and des-gamma-carboxyprothrombin, although their sensitivity and specificity remain limited [4]. Recently, Wang et al. [5] identified an association between cuproptosis and HCC prognosis and developed a cuproptosis-related prognostic signature of treatment response.

Dysregulation in the homeostasis of trace elements, including zinc (Zn), copper (Cu), and selenium, has also been linked to HCC through mechanisms involving oxidative stress, DNA damage, cell cycle progression, and angiogenesis [6]. Moreover, these trace elements may influence the tumor microenvironment and the balance of other trace elements, and novel types of cell death, including ferroptosis and cuproptosis, have recently been associated with hepatocarcinogenesis [7].

Small HCC is characteristically visualized on magnetic resonance imaging (MRI) as a hyperintense mass relative to the surrounding liver parenchyma on T1-weighted images [8]. In approximately one-third of HCC cases exhibiting this pattern, the high signal intensity can be attributed to steatosis [9] or to excessive accumulation of Cu and Zn within the tumor [10]. However, the significance of Cu accumulation remains controversial due to inconsistent findings.

Synchrotron radiation–excited X-ray fluorescence (SR-XRF) imaging has emerged as a novel non-destructive, multi-element subcellular imaging method. It uses monochromatic synchrotron radiation as an X-ray source and a high-quality Fresnel zone plate, enabling the acquisition of high spatial resolution images of trace element distribution [11,12].

Compared with other subcellular imaging methods, including electron energy loss spectroscopy, electron-probe X-ray microanalysis, and proton-induced X-ray emission, SX-RF–based techniques can image thick tissue sections with high spatial resolution and provide high elemental sensitivity. These features enable the visualization of the distribution of many essential cellular metals in situ with high sensitivity [13].

Although the usefulness of SR-XRF for studying the distribution of elements, including Zn and Gd, has been demonstrated in animal models of cancer [14], studies on its prognostic and diagnostic application in HCC remain scarce.

We hypothesized that intratumoral Cu accumulation reflects tumor differentiation status and should be interpreted primarily as a biological marker rather than a direct physical determinant of MRI signal intensity. This study aimed to investigate the utility of SR-XRF for analyzing trace metal distribution in HCC, specifically comparing Cu and Zn distribution obtained with SR-XRF to findings from histopathology and MRI.

## 2. Materials and Methods

This case–control study included 33 specimens from 32 patients (27 males, 5 females; age range, 30–79 years; mean age, 61.5 ± 12.1 years [standard deviation, SD]) diagnosed with HCC, who underwent surgical resection (*n* = 29) or biopsy (*n* = 3) at Kobe University Hospital between December 1999 and November 2002. An appropriate institutional review board approved this study. All patients provided informed consent, and all procedures were conducted in accordance with the ethical standards of the responsible committee on human experimentation (institutional and national) and with the Helsinki Declaration of 1975, as revised in 2008.

All patients had chronic hepatitis or liver cirrhosis, associated with hepatitis B surface antigen in 6 (19%) patients, HCV in 24 (75%), alcohol in 1 (3%), and unknown etiology in 1 (3%).

Thirty-three surgically resected or biopsy specimens, including HCC and surrounding liver parenchyma, were evaluated. HCC was diagnosed histologically on hematoxylin and eosin-stained sections according to the World Health Organization (WHO) criteria [15]. A hepatic pathologist evaluated the degree of histologic differentiation. We expanded the description of histopathological criteria for tumor differentiation based on the WHO classification [15] and standard pathological evaluation to improve transparency and reproducibility.

### 2.1. Sample Preparation

For SR-XRF imaging, specimens of HCC and surrounding liver parenchyma were fixed in 20% buffered formalin (pH 7.2) and embedded in paraffin. Semithin sections, 5 µm thick, were cut from paraffin blocks and mounted on polyimide films (Kapton; Toray Co., Ltd., Tokyo, Japan).

### 2.2. SR-XRF Imaging Setup

**Figure 1:**
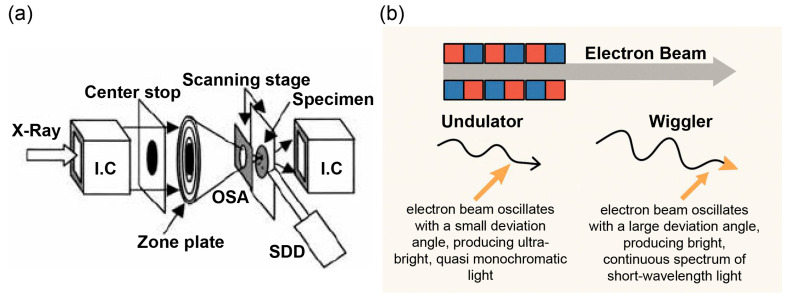
(**a**) Optical system of the microscope. Monochromatic X-rays at 10 keV were focused onto a specimen by a Fresnel zone plate (outermost zone width, 0.1 mm; diameter, 155 mm). The first-order focus was used, and other-order X-rays were eliminated by an order-sorting aperture (OSA, diameter, 20 mm). Transmission and fluorescence X-rays were recorded using an ionization chamber and a silicon drift detector (SDD; Rontec Corp., Stendal, Germany), respectively. This schematic illustrates magnet arrangements and radiation paths in the BL47XU beamline at SPring-8. (**b**) Synchrotron radiation is emitted from insertion devices located in straight sections of the electron orbit. Undulator: small-angle oscillations generate ultra-bright, quasi-monochromatic light by interference. Wiggler: large-angle oscillations yield intense, broad-spectrum light.

Synchrotron radiation is emitted from an insertion device comprising magnet rows with alternating polarity, installed in a straight section of the electron orbit. Depending on magnetic field strength, the following two types of insertion devices can be used: the undulator, where the electron beam wiggles with a small deviation angle, producing ultra-bright and quasi-monochromatic light through interference effects; and the wiggler, where the electron beam wiggles with a large deviation angle, generating bright, spectrally continuous light with shorter wavelengths (Figure 1a,b).

The X-ray experiment was performed using the beamline undulator BL47XU at the SPring-8 synchrotron radiation facility (Hyogo, Japan), a third-generation synchrotron radiation facility that provides highly intense synchrotron radiation. The undulator gap was set at 14.35 mm to provide the first-order harmonics peak at approximately 10 keV. The excitation energy of 10 keV was selected to optimize fluorescence yield for Cu and Zn while minimizing background noise. For submicrometric X-ray beam experiments, a 10 µm horizontal slit was placed before a tantalum Fresnel zone plate. The fluorescent X-rays were analyzed using an energy-dispersive detector (Figure 1 and Figure S1). Two-dimensional mapping of Cu and Zn was performed by raster scanning the specimens. Images ranged from 50 × 50 to 275 × 275 pixels, with 1.0 µm spatial resolution and a measurement time of 0.2 s/pixel. The distribution of Cu and Zn in tumors and surrounding liver parenchyma was measured.

The tumor-to-liver ratio (TLR) of metal content between HCC and liver parenchyma was calculated as follows: TLR = accumulation in tumor ÷ accumulation in surrounding liver parenchyma on SR-XRF images.

### 2.3. MRI

Preoperative MR images were obtained in 28 patients using a 1.5-T superconducting MRI system (Gyroscan ACS-NT/Intera; Philips Medical Systems, Best, The Netherlands) with a synergy body coil. Imaging parameters were: 192 × 256 matrix with a 75% rectangular field of view (28 × 35 cm) and 8 mm slice thickness with no interslice gap. Axial T1-weighted gradient-echo or spin-echo sequences (TR/TE = 150–500/4.4 or 15 ms) and T2-weighted turbo spin-echo sequences (TR/TE = 1500–1800/90 ms) were obtained. Fat suppression was applied to T2-weighted images. Two radiologists classified the signal intensity of HCC relative to surrounding liver parenchyma on T1-weighted imaging (T1WI) and T2WI into three patterns: high, iso, and low.

### 2.4. Data Analysis

Thirty-three HCC specimens from 32 patients were used to compare SR-XRF with histopathology, and 28 specimens from 28 patients were used to compare SR-XRF with MRI.

All analyses were performed using SPSS Statistics for Windows, version 10 (SPSS Inc., Chicago, IL, USA). The relationship between tumor size and TLR of metal content was analyzed using Pearson’s correlation coefficient. The relationships among histologic differentiation, signal intensity on T1WI and T2WI, and TLR of metal content were analyzed using the Wilcoxon rank-sum test or Kruskal–Wallis test. A *p*-value of <0.05 was considered statistically significant.

## 3. Results

**Figure 2:**
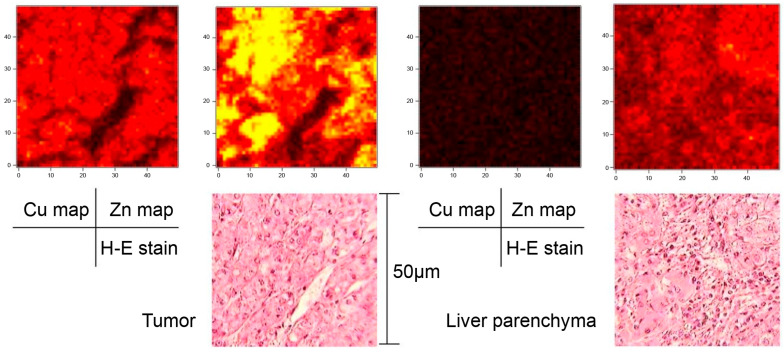
Case 1. A 75-year-old patient with well-differentiated HCC, 8 mm in diameter. The **left** images show the copper and zinc maps of the tumor compared to H&E staining with an area of 50 × 50 µm. The **right** images show the copper and zinc maps of the surround liver parenchyma compared to H&E staining with an area of 50 × 50 µm. These two-dimensional maps of trace metals were obtained using the SR-XRF imaging system. HCC, hepatocellular carcinoma; SR-XRF, Synchrotron Radiation–excited X-ray Fluorescence; H&E, hematoxylin and eosin.

The average tumor diameter was 47 ± 44 mm (SD), ranging from 8 to 150 mm. The SR-XRF imaging system enabled two-dimensional mapping of trace metals with high spatial resolution (1.0 µm). The resulting maps clearly demonstrated the distribution of Cu and Zn at both the intracellular and extracellular levels (Figure 2 and Figure S2).

**Figure 3:**
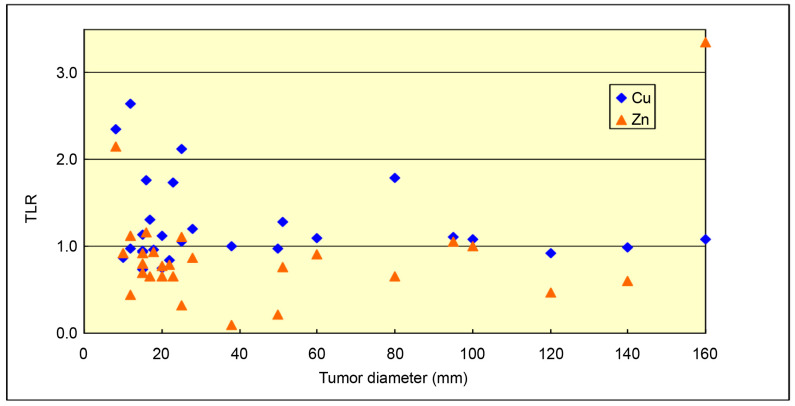
Relationship between mean TLRs of metal content and tumor diameter. No significant correlation was observed. TLR, tumor-to-liver ratio.

No significant correlation was found between tumor diameter and mean TLRs of Cu and Zn (Cu: r = −0.18, *p* = 0.34; Zn: r = 0.31, *p* = 0.09; Figure 3).

**Figure 4:**
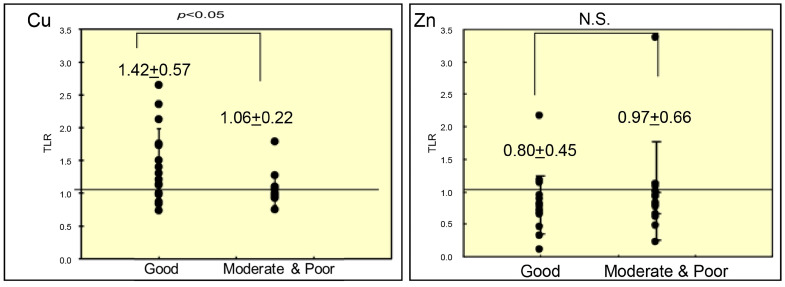
Mean TLRs of copper and zinc content according to histologic differentiation of HCCs (mean ± SD). *p*-values were calculated using the Wilcoxon rank-sum test. TLR, tumor-to-liver ratio; HCC, hepatocellular carcinoma; SD, standard deviation; N.S., not significant.

Figure 4 shows the relationship between the mean TLRs of metal content and histologic differentiation of the tumors. The mean TLRs of Cu content were significantly higher in well-differentiated HCCs than in moderately or poorly differentiated HCCs (1.42 ± 0.57 vs. 1.06 ± 0.22; *p* < 0.05). No significant differences were observed in the mean TLRs of Zn content between well-differentiated and moderately/poorly differentiated HCCs.

**Figure 5:**
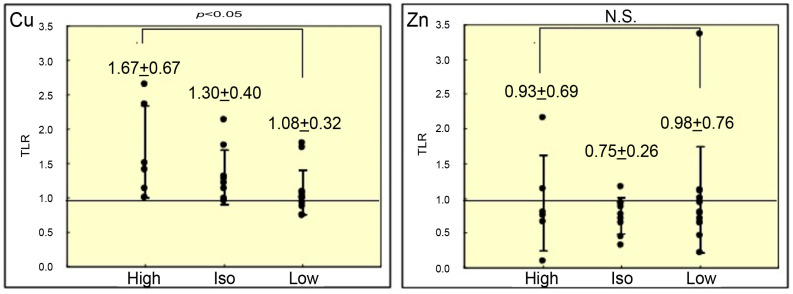
Mean TLRs of copper and zinc content according to signal intensity on T1WI (mean ± SD). *p*-values were calculated using the Kruskal–Wallis test. TLR, tumor-to-liver ratio; SD, standard deviation; N.S., not significant.

Figure 5 shows the relationship between the mean TLRs of metal content and T1WI intensity. The mean TLRs of Cu content were significantly higher in hyperintense lesions than in iso- or hypointense lesions on T1WI (1.67 ± 0.67 vs. 1.30 ± 0.40 vs. 1.08 ± 0.32, respectively; *p* < 0.05). No significant differences were observed in the mean TLRs of Zn content among lesions with different intensities.

**Figure 6:**
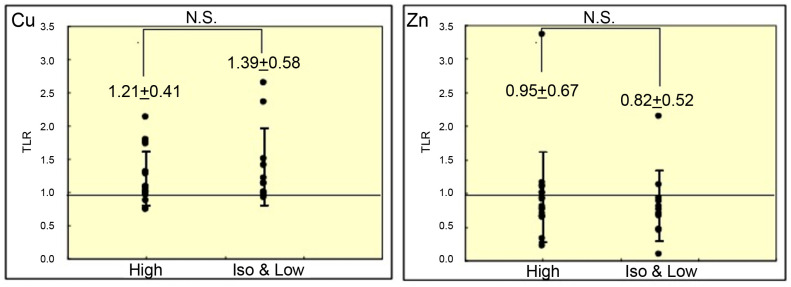
Mean TLRs of copper and zinc content according to signal intensity on T2WI (mean ± SD). *p*-values were calculated using the Wilcoxon rank-sum test. TLR, tumor-to-liver ratio; SD, standard deviation; N.S., not significant.

Figure 6 shows the relationship between the mean TLRs of metal content and T2WI intensity. No significant differences were found in mean TLRs of Cu or Zn content between hyperintense and iso-intense/hypointense lesions on T2WI.

An illustrative case of a patient with well-differentiated HCC is presented (see Figure S3, which presents an illustrative case (Case 5) of a well-differentiated HCC, 15 mm in diameter, with TLR (Cu and Zn) values of 2.65 and 0.43, respectively). On both in-phase and opposed-phase T1-weighted images, this HCC appeared as a hyperintense area, whereas on T2-weighted images, it appeared isointense. Additionally, SR-XRF images of the peripheral region of this nodule clearly demonstrated differences in Cu and Zn distribution between the tumor and surrounding liver parenchyma.

## 4. Discussion

We investigated the usefulness of SR-XRF for studying trace metal distribution in HCC, particularly comparing Cu and Zn distribution in SR-XRF with histopathological and MRI findings.

Our findings present, for the first time, the visualization of metal dynamics in the tumor microenvironment by correlating elemental distribution maps with histological findings in the same specimen using SR-XRF.

The lack of correlation between tumor size and Cu/Zn TLR may be attributed to tumor heterogeneity and metabolic reprogramming. However, we found that TLRs of Cu content were significantly higher in well-differentiated HCCs than in moderately and poorly differentiated HCCs. This result is consistent with that of a previous study [16], which demonstrated higher accumulation of Cu in small HCCs than in surrounding liver parenchyma, particularly in association with metallothioneins. This suggests that intratumoral Cu content is related to the stepwise carcinogenesis of HCC. Indeed, metallothioneins and their link with DNA methylation have recently been associated with HCC progression and prognosis [17,18].

The finding that Cu TLR is higher in well-differentiated HCC supports the hypothesis that abnormalities in metal metabolism occur during stepwise carcinogenesis and provides a rationale for positioning Cu as a biological indicator of tumor differentiation. Our results also corroborate and expand previous research by Skalny et al. [19], who reported imbalances of Cu in colorectal cancer tissue.

By providing quantitative evidence of Cu accumulation in well-differentiated HCC, our study reinforces conventional pathological and analytical chemical findings with spatially resolved information. Moreover, the observed association between intratumoral Cu accumulation and T1WI hyperintensity provides elemental-level evidence that may help interpret MRI signal characteristics; however, this relationship should be interpreted cautiously and not as proof of causality. We also found that mean TLRs of Cu content were significantly higher in hyperintense HCCs than in iso- or hypointense HCCs on T1WI, suggesting that intratumoral Cu influences MRI signal intensity. Metals in HCCs have been considered to exert paramagnetic effects, implying that intratumoral Cu or Zn may alter MRI signal intensity [8,20]. Importantly, well-differentiated (early) HCCs frequently contain intratumoral fat (steatosis), which is a well-established source of T1-weighted hyperintensity. Therefore, the T1 signal in such lesions may originate predominantly from fat, with Cu accumulation reflecting retained hepatocyte-like metabolic features and serving primarily as a marker of differentiation rather than a dominant physical driver of T1 shortening. In this cohort, routine quantitative fat assessment (e.g., chemical-shift–based fat fraction or proton density fat fraction [PDFF]) was unavailable and T1-weighted signal intensity was classified visually; thus, residual confounding by steatosis cannot be excluded. Future studies integrating SR-XRF metrics with standardized fat quantification (in-/opposed-phase signal drop or PDFF) and histologic steatosis grading will be crucial to disentangle the relative contributions of fat and trace metal accumulation to T1 signal characteristics. This relationship has been examined using staining methods, metal-binding protein staining, atomic absorption spectrophotometry, and particle-induced X-ray emission (PIXE) analysis. However, staining methods (e.g., orcein staining) were found to be unreliable for quantifying metal content [10,21,22,23,24,25]. These limitations were partly addressed with spectrophotometry and PIXE analysis, which can quantify metal content independently of distribution [16]. Our study underscores the usefulness of SR-XRF for simultaneously investigating the distribution and concentration of trace metals in HCC [26,27,28].

Overall, our results suggest a relationship among Cu accumulation, tumor differentiation, and T1WI hyperintensity. These findings support the potential of Cu TLR as a marker of tumor differentiation, contributing to diagnosis, prognosis estimation, and interpretation of stepwise tumor progression, ultimately bridging imaging, pathology, and elemental omics.

Several studies have reported altered distribution and quantity of trace metals (Zn, Cu, Fe, and Cs) across cancers. For example, Planeta et al. [29] showed that Fe, Cu, and Se may serve as biomarkers of glioblastoma progression, while Udali et al. [18] demonstrated that high serum Cu levels were directly associated with decreased survival. In fact, patients in the highest quintile of serum Cu had a sixfold greater mortality risk than those in other quintiles. In contrast to Cu—which may accumulate in well-differentiated tumors reflecting retained hepatocyte-like metabolic features—Zn may remain relatively stable at the tissue level because it is an essential cofactor with stringent homeostatic control. Thus, Zn-related alterations in HCC may preferentially manifest as redistribution (subcellular/compartmental shifts) rather than a net increase measurable by mean TLR, potentially explaining the absence of significant Zn associations in this analysis.

Recently, SR-XRF has emerged as a novel non-destructive, multi-element method that uses monochromatic synchrotron radiation as an X-ray source and a high-quality Fresnel zone plate, enabling high-resolution imaging of trace element distribution [11,12]. By allowing non-destructive, multi-element, high-sensitivity mapping, this technique integrates “distribution” and “quantification,” which were difficult to achieve with conventional methods, including staining and bulk quantification. It enables the evaluation of tumor–liver boundaries and tumor heterogeneity. However, it should be noted that SR-XRF is positioned as a complementary ex vivo element imaging technique, not as a competing clinical imaging diagnostic tool, including positron emission tomography (PET).

The ability to apply this method to fixed and embedded specimens enhances its compatibility with pathological workflows, supporting potential future applications in pathological diagnosis and treatment response evaluation based on element dynamics.

This study had some limitations. First, it was a single-center study with a limited number of cases, and outcomes were measured in a cohort enrolled between 1999 and 2002. Therefore, caution is warranted regarding external validity and generalizability to current treatments and imaging conditions. Second, challenges remain in controlling the effects of fixation and embedding on element quantification; the standardization of region-of-interest definitions and colocalization analysis is necessary for accurate TLR calculation. Third, access to synchrotron facilities and throughput remain bottlenecks for clinical applications. Finally, formalin fixation and paraffin embedding processing may alter bulk metal concentrations and/or lateral elemental distributions via diffusion, solvent-related washout, and tissue shrinkage; these effects are known to be element- and tissue-dependent. Therefore, our Cu/Zn TLR results should be interpreted cautiously, and future studies using paired fresh-frozen specimens are warranted [30,31,32,33].

## 5. Conclusions

To the best of our knowledge, this is the first report of SR-XRF applied to human HCC. By comparing high-resolution elemental maps with histopathology, SR-XRF provides important insights into the pathophysiological processes of tumor formation and progression. Although its use in HCC research is still exploratory, this technique has potential for clinical application. Future directions include tumor characterization, personalized treatment monitoring, and the identification of novel biomarkers, contingent upon further technical refinements—including multi-element mapping and integration into pathology workflows—and large-scale validation studies.

## Data Availability

The datasets generated during and/or analyzed during the current study are not publicly available, but are available from the corresponding author on reasonable request.

## Supplementary Materials

The following supporting information can be downloaded at https://www.mdpi.com/article/10.3390/cancers18020311/s1, Figure S1: Experimental setup of SR-XRF imaging. The thin polyimide film with the specimen was inclined 45° from the X-ray beam path. The SDD was positioned 90° from the beam path to detect fluorescent X-rays with minimal contribution from elastic scattering. The sample was scanned in two dimensions; Figure S2: (a–f) Two-dimensional maps of trace metals obtained by the SR-XRF imaging system. All images were 275 × 275 pixels with 1.0 µm spatial resolution. (g–i) Magnified photographs reveal scanning areas of moderately differentiated HCC, well-differentiated HCC, and surrounding liver parenchyma, respectively, in a patient (Case 2) with a nodule-in-nodule type HCC, consistent with stepwise hepatocarcinogenesis; Figure S3: This case is a patient with well-differentiated HCC. On both in-phase and opposed-phase T1-weighted images, the lesion appears as an area of high intensity, while the T2-weighted image shows it as an area of iso intensity. Additionally, XR-SRF images of the peripheral area of this nodule clearly indicate the differences in the copper and Zn distributions between the tumor and surrounding liver parenchyma.

## Abbreviations

The following abbreviations are used in this manuscript:

AFP	alpha-fetoprotein
HCC	hepatocellular carcinoma
HCV	hepatitis C virus
MRI	magnetic resonance imaging
OSA	order-sorting aperture
PDFF	proton density fat fraction
PIXE	particle-induced X-ray emission
SDD	silicon drift detector
SR-XRF	Synchrotron Radiation–excited X-ray Fluorescence
TLR	tumor-to-liver ratio
WHO	World Health Organization
