# Supplementary figures and images for: Synchrotron Radiation–Excited X-Ray Fluorescence (SR-XRF) Imaging for Human Hepatocellular Carcinoma Specimens
Source: Cancers (Basel). 2026 Jan 20;18(2):311. doi: 10.3390/cancers18020311 (PMC12839296; doi:10.3390/cancers18020311)

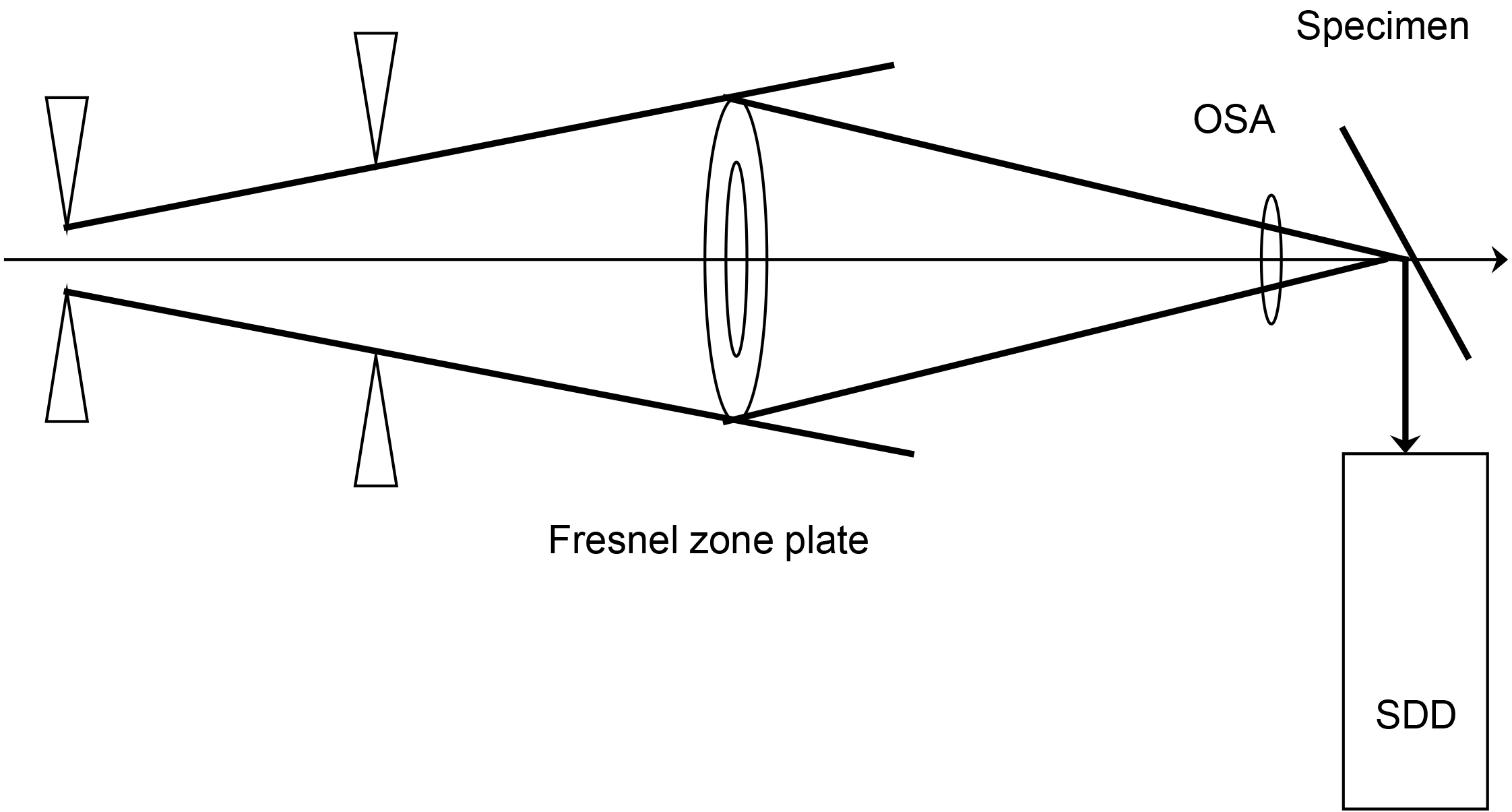

Supplement: Supplementary file 1 [file cancers-18-00311-s001.zip › Figure S1.tif]

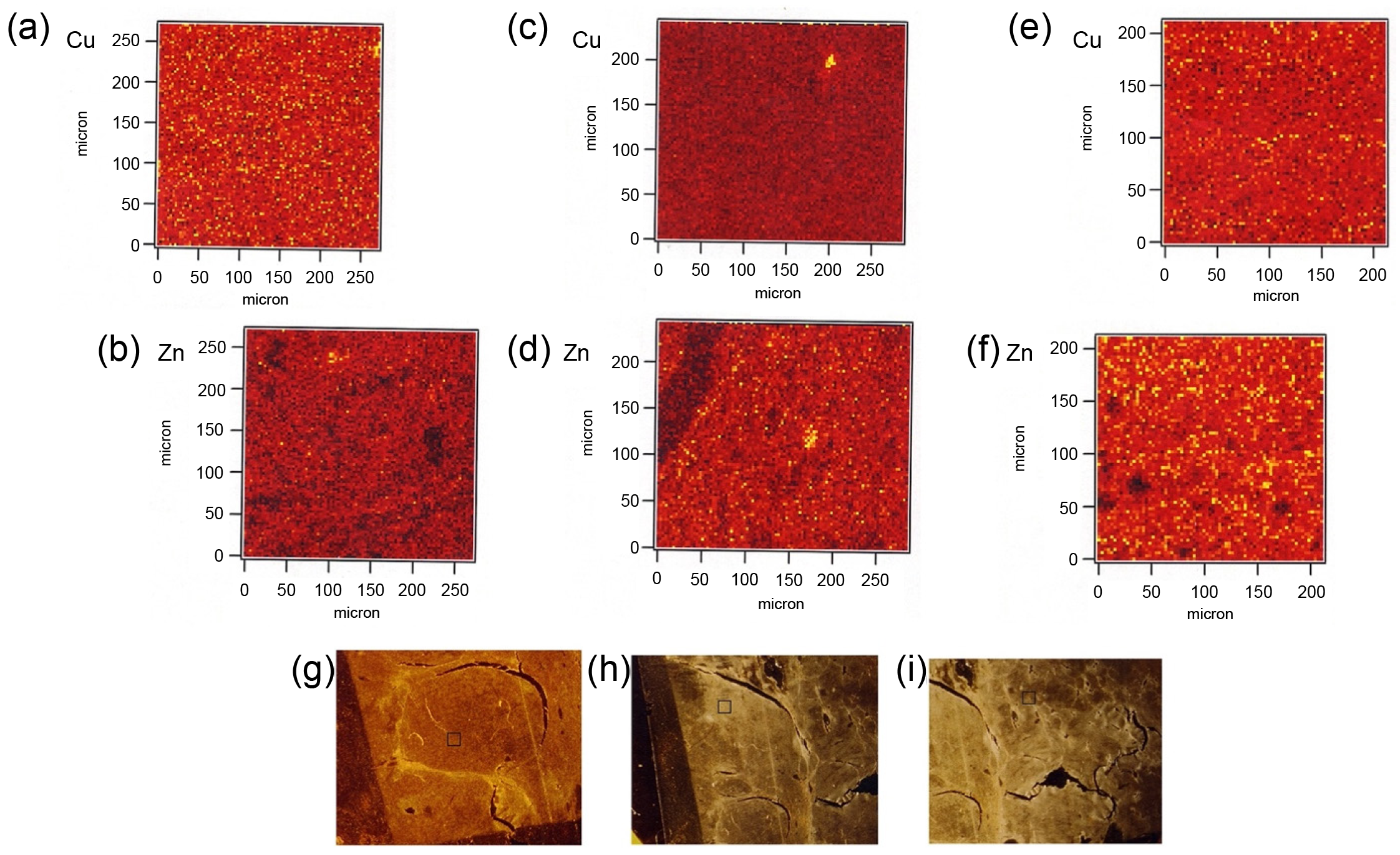

Supplement: Supplementary file 1 [file cancers-18-00311-s001.zip › Figure S2.tif]

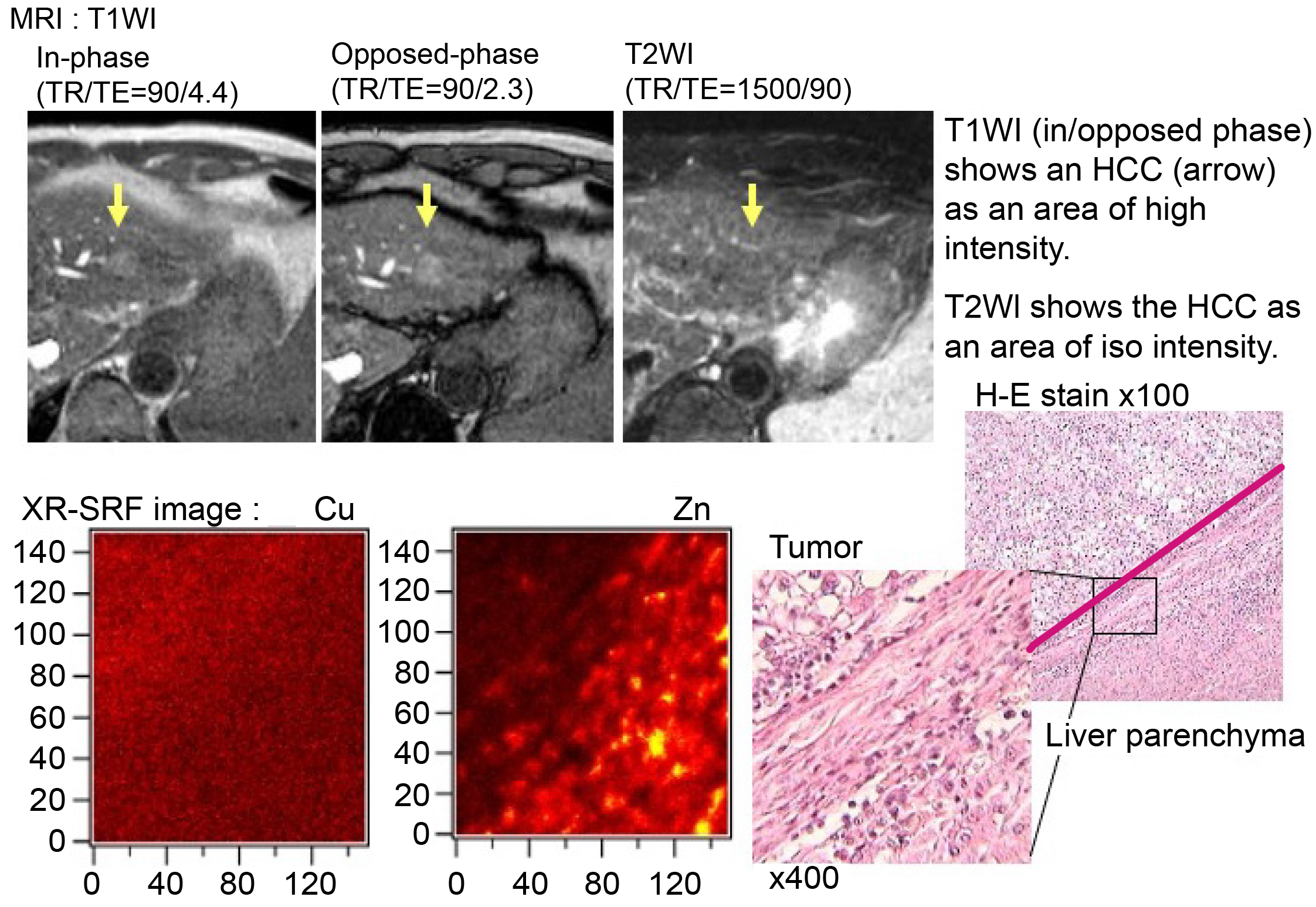

Supplement: Supplementary file 1 [file cancers-18-00311-s001.zip › Figure S3.tif]
